# Are Microplastic (∼25–1000 μm) and Plasticizer Concentrations Correlated in Sediments of an Urbanized UK Estuary?

**DOI:** 10.1021/acs.est.5c12721

**Published:** 2025-12-04

**Authors:** Alex Billings, Richard K. Cross, Francis Daunt, Justyna P. Olszewska, Amy Pickard, Maria I. Bogdanova, Ruairidh Cox, Kevin C. Jones, David J. Spurgeon, M. Glória Pereira

**Affiliations:** † 41865 UK Centre for Ecology & Hydrology, Library Avenue, Bailrigg, Lancaster LA1 4AP, United Kingdom; ‡ Lancaster Environment Centre, 4396 Lancaster University, Lancaster, LA1 4YQ, United Kingdom; § UK Centre for Ecology & Hydrology, Benson Lane, Crowmarsh Gifford, Wallingford OX10 8BB, United Kingdom; ⊥ UK Centre for Ecology & Hydrology, Bush Estate, Penicuik EH26 0QB, United Kingdom

**Keywords:** plasticizers, phthalates, sediments, estuaries, microplastics, emerging contaminants, plastics

## Abstract

An understanding of the relationships between plastics and plasticizers is vital in order to assess their environmental risk. We investigated spatial trends and relationships between microplastics and plasticizers in sediments of an urbanized estuary subject to contemporary and historic sources of contamination (Forth estuary, Scotland, UK). As such, this study represents one of the first to investigate the co-occurrence of emerging plasticizers, phthalates, and microplastics in an estuary system. We determined the concentration of 7 legacy (phthalate) and 3 emerging (adipate, terephthalate, trimellitate) plasticizers and 21 microplastic polymer types. The most abundant microplastics were polyethylene (PE), polypropylene (PP), polyurethane (PU), and poly­(vinyl chloride) (PVC). Plasticizers were dominated by diethylhexyl phthalate (DEHP), although emerging plasticizers (e.g., diethylhexyl terephthalate, DEHTP) were frequently detected at low concentrations (mean 7.3 ng g^–1^ ww). There was strong evidence that concentrations of microplastics and plasticizers were significantly lower in the outer estuary. However, we found no evidence for a spatial relationship between the concentration of microplastics and plasticizers at individual site level. Our results indicate that microplastics in the size range analyzed (∼25–1000 μm) may not be a good predictor of the spatial distribution of plasticizers in estuaries. This could result from release of plasticizers prior to plastic fragmentation and deposition and differences in transport and fate.

## Graphical abstract

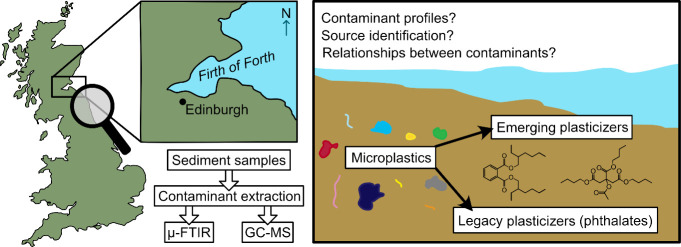

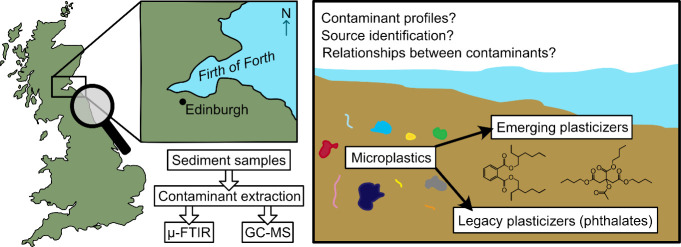

## 1 Introduction

Microplastics can be considered plastic materials <5 mm in size, either intentionally produced^1^ or generated through degradation and fragmentation of larger items.^2^ Considerable efforts are being made to understand exposure to microplastics from environmental and human health perspectives,^3,4^ with concerns raised by the observation of around 1500 species documented to ingest microplastic particles.^5^ As well as the polymers themselves, microplastics are also composed of a range of additives. One of the most widely produced class of additives are plasticizers, which can typically constitute 10–70% w/w of the plastics in which they are used.^6^ The occurrence of plasticizers is receiving increasing research attention in freshwater^7^ and marine^8^ environments due to concerns surrounding their uptake and effects on a range of taxa, e.g., hepatic stress and genotoxicity in fish^9,10^ and oxidative stress in seabirds.^11^

Although the majority of plastics and plasticizers are used on land, these contaminants may be transported to freshwater environments through, e.g., wastewater treatment plant (WWTP) outflows, sewage sludge runoff from agricultural land, and diffuse releases from urban or industrial areas. They can then be transported to estuaries which are sites of extensive sediment deposition and therefore may act as medium-term sinks for microplastics and plasticizers. Concerns surrounding the uptake and impacts of these contaminants further highlight that understanding estuarine occurrence and fate of microplastics and plasticizers is key to assessing their environmental risk. In particular, it is important to consider whether the fate of plasticizers associated with disintegrating plastic material is driven by microplastic transport dynamics or whether dissociation dynamics can lead to exposure to “free” plasticizer molecules.

Previous studies have reported widespread microplastic contamination of estuaries,^12–14^ although reported concentrations span several orders of magnitude,^15^ likely in part due to differences in analytical methods used. Knowledge of occurrence of phthalate plasticizers in estuarine sediments is more limited.^16,17^ Additionally, there is very little data concerning the occurrence of emerging plasticizers increasingly used as phthalate replacements.^18^ Consequently, the co-occurrence of microplastics and phthalate and emerging plasticizers in estuarine sediments has not been well characterized.

To address this gap, the aim of this study was to investigate the spatial patterns of occurrence of phthalate and emerging non-phthalate plasticizers and microplastics in sediments of the Firth of Forth, an urbanized estuary in Scotland, UK. A number of potential sources of these contaminants are located along the estuary (Figure 1), including landfill sites, municipal and industrial WWTP outfalls, and urban areas with diffuse sources of plasticizers, e.g., linked to the city of Edinburgh. The Forth estuary also has a complex topology of depths and sediment types and exhibits a large seasonal variation in flow condition,^19^ making the estuary an excellent location to study the distribution of, and relationships between, microplastics, phthalates, and emerging plasticizers in estuarine sediments.

**Figure 1:**
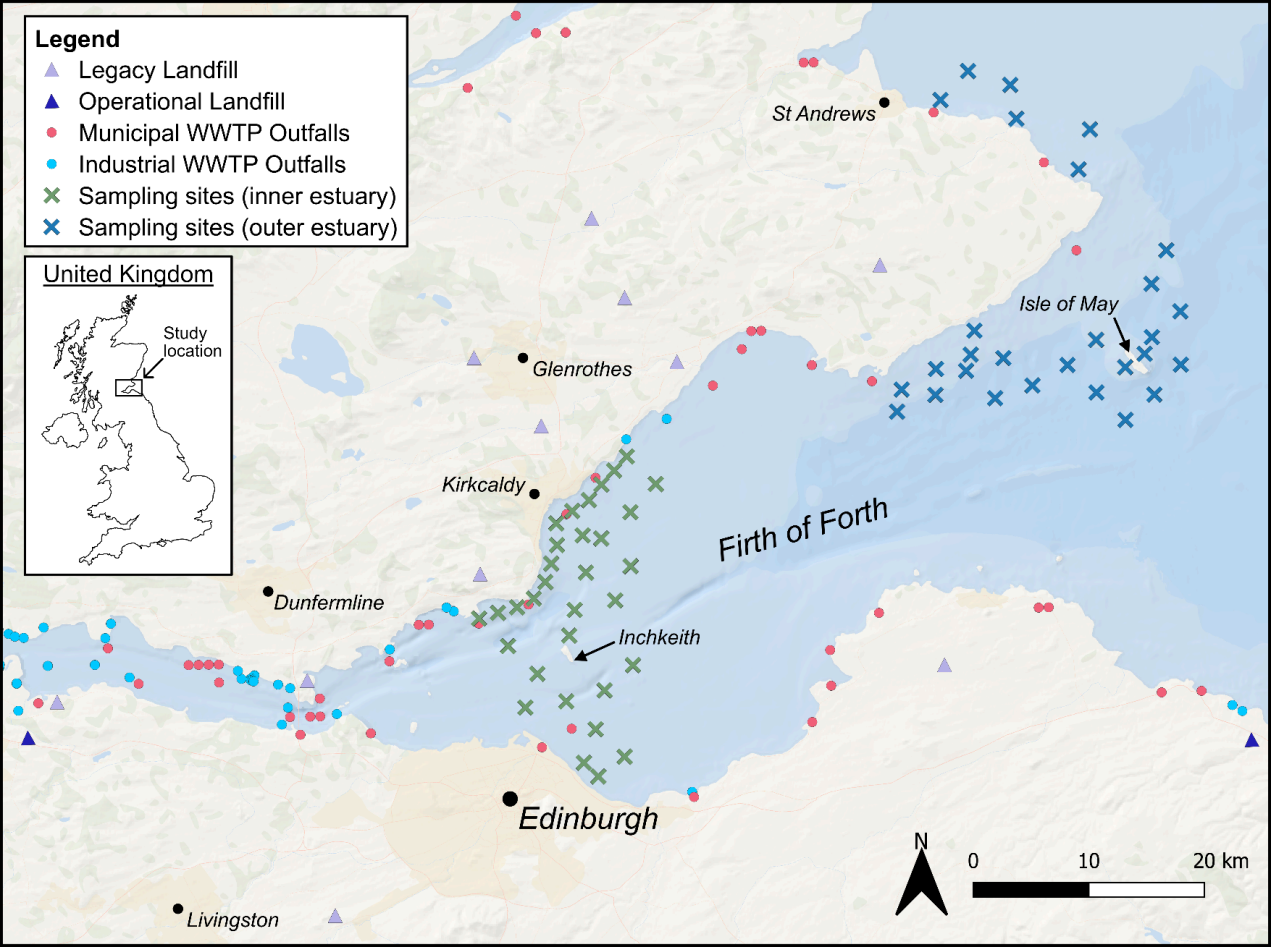
Map of coastal landfill sites, wastewater treatment plants (WWTPs), and sediment sampling sites in the Firth of Forth estuary in Scotland, UK; landfill sites within 10 km of the Forth estuary and/or within 5 km of a tributary are shown; contains information from the public sector licensed under the Open Government License v3.0.

Sediments in the Forth estuary were sampled from two areas representing high and low proximities to potential contaminant sources. We used GC-MS (gas chromatography–mass spectrometry) to determine the concentration of phthalate and emerging plasticizers in the sediments. Micro Fourier transform infrared spectroscopy (μ-FTIR) was used to quantify abundance of microplastics (≥25–1000 μm in diameter). This is the first study we are aware of to investigate co-occurrence of emerging plasticizers, legacy plasticizers, and microplastics in estuaries. The analyses conducted allowed us to test three hypotheses relating to microplastic and plasticizer levels: (1) Contaminant concentrations will be related to source proximity with greater abundance at the inner estuary than at the outer estuary. (2) Profiles of plasticizers and microplastics will reflect historic and current use patterns. (3) Plasticizer concentrations will be correlated with microplastic abundance.

## 2 Materials and Methods

### 2.1 Study Area and Sample Collection

The Firth of Forth is an estuary in Scotland, UK. The major settlements along the estuary have a total population of ∼630,000.^20^ Average annual channel flow is 63 m^3^ s^–1^, ranging from <10 m^3^ s^–1^ in summer to >300 m^3^ s^–1^ in winter.^19^ There are a number of legacy and operational landfill waste sites along the course of the estuary in addition to outfalls from wastewater treatment plants (WWTPs) treating public sewerage and waste from manufacturing plants (Figure 1).^21,22^

85 bed sediment samples were collected from November 2020 to July 2021 from sites in the inner estuary (sites west of longitude −2.9; Figure 1, green crosses) and the outer estuary (sites east of longitude −2.9; Figure 1, blue crosses). Selection of these two areas was based on differences in distance from putative sources of microplastics and plasticizers (Figure 1). A 250 cm^2^ Van Veen grab sampler was used to collect samples from the surface of the estuary bed at a range of water column depths (2–57 m). Samples were stored in glass containers at −20 °C. Samples were collected at three time periods, winter (*n* = 53; inner = 26; outer = 27; 03.11.20–03.03.21), spring (*n* = 19; inner = 7; outer = 12; 07.04.21–09.06.21), and summer (*n* = 13; inner = 13; 14.07.21–23.07.21), from a total of 60 sites in the estuary and neighboring coastal area (Figure 1). Samples in summer were collected from the inner estuary only due to logistic challenges. 58 samples representing 45 sites were analyzed for microplastics and plasticizers (inner = 28, outer = 30; winter = 39, spring = 13, summer = 6). Due to limited sample availability, 12 samples were analyzed for microplastics only (inner = 6, outer = 6; winter = 6, spring = 3, summer = 3), and 15 for plasticizers only (inner = 12, outer = 3; winter = 8, spring = 3, summer = 4).

### 2.2 Microplastic Extraction and Analysis

Sample preparation for microplastic analysis followed a method previously described.^23^ Briefly, sediment was mixed thoroughly with a cleaned stainless steel spatula for subsampling. ∼30 g wet weight (ww) was used for sample preparation to ensure sufficient extracted material to be above limits of detection (LODs). The dry weight (dw) of sediments was measured separately from a concurrent subsample. A Fenton’s reaction maintained at <50 °C was performed to oxidize organic material (10 mL of Fe­(II) 0.05 M solution and 20 mL of >30% H_2_O_2_). Density separation was performed overnight on digested solids suspended in ZnCl_2_ at 1.7 g cm^–3^ in 100 mL cylinders. The supernatant containing floated microplastics was decanted, and the ZnCl_2_ column was mixed with fresh ZnCl_2_ to perform a second extraction.^24^ Combined supernatants were filtered for a second Fenton’s reaction. A size separation into coarse (>198 μm) and fine (<198 μm) fractions, both of which were analyzed and data were combined, prevented overloading of analysis filters. Samples were stored in 50% ethanol before depositing and analysis.

For spectroscopic μ-FTIR analysis, extracted samples were mixed by vigorously shaking for 10 s; then, the (sub)­sample was immediately deposited onto a silver membrane filter (25 mm diameter; 3 μm pore; Sterlitech, Washington USA). >50% of the material was targeted for deposition.^25^ All microplastics within the deposition area of ∼11 × 11 mm were identified and quantified with an imaging μ-FTIR spectrometer (PerkinElmer Spotlight 400) set to collect spectra between 4000 and 700 cm^–1^. A background spectrum of the silver filter was collected and removed from the resulting data. The pixel size selected was 25 μm, and thus, this was the minimum theoretical particle size that could be quantified (see Appendix S1).

Mapping was carried out at a resolution of 8 cm^–1^ with two scans per pixel and an interferometer speed of 2.2 cm s^–1^. Automated spectral matching of all raw data was performed using the Purency Microplastics Finder. Purency uses a model-based machine learning approach toward polymer identification based on a random decision forest classifier.^26^ The model was applied without any manual correction. Complete verification of the algorithm with a mixture of environmentally relevant standards would be the ideal assessment of the model. To approximate this, recovery was successfully demonstrated for two relevant microplastics (polystyrene and polyamide) in blank samples to confirm the correct classification of microplastics in samples. Baselines for background contamination not associated with the samples were established through a program of blank samples run alongside each batch of samples to establish the limits of detection. The performance of this model has also been tested, and examples of its application include samples from deep sediments.^27^ While there are several automated pipelines available for spectral analysis of microplastics, a single approach provides consistency in measurement over manual approaches. The output generates particle counts by polymer type for 21 common plastic polymers and provides information on two-dimensional aspects of the particles. These polymers are polypropylene (PP), polyethylene (PE), poly­(vinyl chloride) (PVC), polyurethane (PU), polyethylene terephthalate (PET), polystyrene (PS), acrylonitrile butadiene styrene (ABS), polyamide (PA), polycarbonate (PC), poly­(methyl methacrylate) (PMMA), polyoxymethylene (POM), cellulose acetate (CA), ethylene-vinyl-acetate copolymer (EVAc), ethylene vinyl alcohol (EVOH), polyacrylonitrile (PAN), polybutylene terephthalate (PBT), polyether ether ketone (PEEK), polyphenylsulfone (PPSU), polysulfone (PSU), silicone, and poly­(lactic acid) (PLA).

Data was blank-corrected (using mean per polymer in the blanks) and reported only if >LOD on a polymer-by-polymer basis. LOD was defined as 3.3× standard deviation of blanks (*n* = 6).^28,29^ Process losses of microplastics to equipment during the extraction procedure was tested through recovery assessment in blank water samples alongside sample batches with a suspension of polyamide (PA, 60–93 μm, dry powder produced in house through cryo-milling) and polystyrene microplastics in water (PS, 45 μm, liquid dispersion, Polysciences Europe GmbH, Germany) to confirm method performance (*n* = 5). Recovery of PS was 95% (RSD 17%) and of PA was 80% (RSD 60%). Quality assurance and quality control are summarized in Figure S1. Microplastic data is reported on a dw basis, except in cases where comparisons are made to plasticizer data (ww).

### 2.3 Plasticizer Extraction and Analysis

A suite of 7 legacy (phthalate) and 3 emerging plasticizers were measured in the samples (Table S1). Specifically, dimethyl phthalate (DMP), diethyl phthalate (DEP), di-iso-butyl phthalate (DiBP), di-*n*-butyl phthalate (DnBP), benzyl butyl phthalate (BBP), diethylhexyl phthalate (DEHP), di-*n*-octyl phthalate (DnOP), diethylhexyl adipate (DEHA), diethylhexyl terephthalate (DEHTP), and trioctyl trimellitate (TOTM) were analyzed. These compounds were selected to provide a representative range of physiochemical parameters potentially relevant to plasticizer fate (e.g., molecular weight, logK_OW_, solubility, chain branching) and because they are known to be commonly used as plastic additives. Plasticizers were extracted according to a method previously described.^30^ In brief, 1.5–3.0 g ww of sediment was homogenized and dried with anhydrous sodium sulfate. Samples were then spiked with deuterium-labeled recovery standards (d4-DnBP and d4-DnOP, Sigma-Aldrich, USA). Analytes were extracted for 30 min using an Ethos X microwave extraction system (Milestone, Italy) with 9:1 dichloromethane:acetone. Supernatants were collected and dried with anhydrous sodium sulfate. Extracts were reduced to a known volume, passed through a PTFE filter (pore size of 0.45 μm), and cleaned using size-exclusion chromatography (Agilent 1200 series HPLC, Agilent, USA). Deuterium-labeled internal standards (d4-DEP and d4-DEHP, Sigma-Aldrich, USA) were added to all samples prior to instrumental analysis.

Analysis was carried out using a GC-MS instrument (6890N-5975B, Agilent, USA) in electron ionization mode. 1.7 μL of sample was injected in splitless mode onto an HP-5ms analytical column (30 m length, 0.25 μm film thickness, 0.25 mm internal diameter, Agilent, USA). Helium was used as the carrier gas (1.5 mL min^–1^). Inlet and MS source temperatures were 300 and 230 °C respectively. LODs were determined from calibration curves of analytical standards (96–99.5% purity; Sigma-Aldrich, USA), mass of sediment analyzed, and dilution factors. LODs ranged from 0.3 to 4.4 ng g^–1^ ww (mean 1.2 ng g^–1^ ww ± 1.4). Recoveries were between 60% and 120% (88% ± 13) and 65% and 119% (100% ± 11) for d4-DnBP and d4-DnOP. Analytes were quantified using internal and recovery standards and calibration curves (see Table S1 for further information). Two procedural blanks were included in each batch of sample extractions. Plasticizer concentrations are reported in ng g^–1^ ww and were recovery and blank-corrected.

### 2.4 Contamination Controls

A full list of contamination controls employed to limit microplastic and plasticizer contamination of the samples is included in the Supporting Information (Appendix S2). These controls were based on those established in previous studies.^28–31^

### 2.5 Data Analysis and Statistical Methods

Microplastic data is reported on a dry weight basis, except in cases where direct comparisons are made to plasticizer data, in which case wet weight concentrations are reported. Microplastic concentrations were converted to wet weight prior to statistical comparison with plasticizers for consistency between contaminant groups. For the calculation of Σplasticizer and Σmicroplastic concentrations for individual sites (and mean and median values), compounds <LOD were assigned a value of zero to avoid overestimation.^32,33^ When carrying out statistical analyses with untransformed data for sites where all analytes were <LOD, Σplasticizer concentration was assigned as 0.5× the mean plasticizer LOD and Σmicroplastic concentration was assigned as 0.5× the median microplastic LOD.^34^ These substitutions were also used prior to any log10-transformation. Median LOD was used for Σmicroplastic due to the much wider range and skewed distribution of LODs for individual microplastic analytes. Statistical analyses were carried out using R (version 4.4.0).^35^

Linear mixed models (LMMs) were used to investigate relationships between Σplasticizer (the sum of all 10 plasticizers) and Σphthalate (sum of the 7 phthalates) concentrations and sampling areas (inner estuary, outer estuary) with season (winter, spring, summer) included as a random effect to control for the fact that sampling occurred across different seasons. Contaminant concentrations were log10-transformed prior to analysis to achieve approximate normality in the distribution of the response variables. LMMs were generated both with and without the 9.6% of samples in the data set which were <LOD for Σplasticizer and Σphthalate, as the posterior predictive check indicated that the LMMs for the full data set deviated from the observed data for those samples in particular (due to the substitution of values <LOD; see above). The conclusions from these comparative analyses were similar (see Section 3.1.). Although the test with <LOD removed is more conservative since the majority of the <LOD samples were located in the outer estuary, outputs from both models are reported in the text where such comparative analyses were carried out.

Generalized linear models (GLMMs) with gamma error structure and log-link function were used to investigate relationships between Σmicroplastic (sum of the 21 microplastic polymers) and Σemerging (sum of the 3 emerging plasticizers) concentrations and sampling areas with the same random effect as the LMMs. We used GLMMs for these contaminants due to better diagnostics than for the same relationships modeled by LMMs (see below for more details about model diagnostics). LMMs were used to model interrelationships between Σplasticizer concentrations and Σmicroplastic and ΣPVC concentrations (on both a mass and count basis) with area and season included as random effects. Contaminant concentrations were again log10-transformed prior to analysis to achieve approximate normality in the distribution of the response variables.

In LMMs and GLMMs, sum concentrations for each contaminant class (e.g., Σplasticizer, Σmicroplastic, etc.) were used as opposed to individual compounds/polymers due to low detection frequencies of some compounds/polymers (see Tables S2 and S3 for full details about the detection frequencies of each analyte).

LMMs and GLMMs were fitted using the “lme4” package.^36^ In cases where response variables exhibited high levels of nondetects (i.e., Σemerging plasticizer concentrations; 42% < LOD), GLMMs accounting for zero-inflation were fitted using the “glmmTMB” package.^37^ *R* ^2^ values for fixed effects in LMMs and GLMMs were estimated following the method established by Nakagawa and Schielzeth^38^ using the package “r2glmm”.^39^ For all models, diagnostic plots were generated using the “performance” package.^40^ Appropriateness of model structure and model validity and accuracy were assessed through comparisons of homogeneity of variance, uniformity of residuals, normality of random effects, influential observations, model linearity (for LMMs), and posterior predictive checks.

Principal component analysis (PCA) was used to investigate patterns of spatial variation and interrelationships between individual plasticizers and microplastics. PCA was performed using individual plasticizer compound and microplastic polymer concentrations, distance of the site from shore, and the sample depth in the water column as input variables. Contaminants with a detection frequency of 0% were removed from the data set. Concentrations that were below the limit of detection were assigned a value of 0.5× LOD. PCA was performed twice using microplastic concentrations on either a mass or a count basis. All input variables were log10-transformed in order to reduce the leverage of outliers in the analysis. Input variables were then mean centered and scaled to unit variance prior to analysis. PCA was carried out using the “FactoMineR” package.^41^

## 3 Results and Discussion

In the following sections, we discuss the three hypotheses that are the focus of this study: (1) Contaminant concentrations will be related to source proximity with greater abundance at the inner estuary than at the outer estuary (Section 3.1). (2) Profiles of plasticizers and microplastics will reflect historic and current use patterns (Sections 3.2 and 3.3). (3) Plasticizer concentrations will be correlated with microplastic abundance (Section 3.4).

### 3.1 The Highest Levels of Plasticizers and Microplastics (∼25–1000 μm in Diameter) Were Found in the Inner Estuary

The data provided very strong evidence that Σmicroplastic concentrations (*R* ^2^_GLMM_ = 0.33, estimate = −1.40, SE = 0.23, *t* = −6.1, *p* < 0.0001) were significantly higher in the inner than the outer estuary (Figure 2; Σmicroplastic mean concentration 5695 vs 1412 particles kg^–1^ ww). There was moderate evidence that Σplasticizer concentrations (*R* ^2^_LMM_ = 0.07, estimate = −0.37, SE = 0.17, *t* = −2.1, *p* = 0.037 [only >LOD]; *R* ^2^_LMM_ = 0.14, estimate = −0.68, SE = 0.20, *t* = −3.4, *p* = 0.001 [all data]) were higher in the inner estuary (Figure 2; Σplasticizer 332 vs 73.5 ng^–1^ ww). Spatial patterns observed for Σplasticizer were also largely reflected by those of the legacy and emerging compound groups. Specifically, while mean Σphthalate and Σemerging plasticizer concentrations were higher in the inner than outer estuary, LMMs indicated that this constituted relatively weak evidence of an effect of sample area on Σphthalate (*R* ^2^_LMM_ = 0.05, estimate = −0.39, SE = 0.21, *t* = −1.9, *p* = 0.067 [only >LOD]; *R* ^2^_LMM_ = 0.11, estimate = −0.66, SE = 0.22, *t* = −3.0, *p* = 0.004 [all data]) and there was little evidence of a significant effect on Σemerging (estimate = −0.42, SE = 0.29, *z* = −1.4, *p* = 0.15).

**Figure 2:**
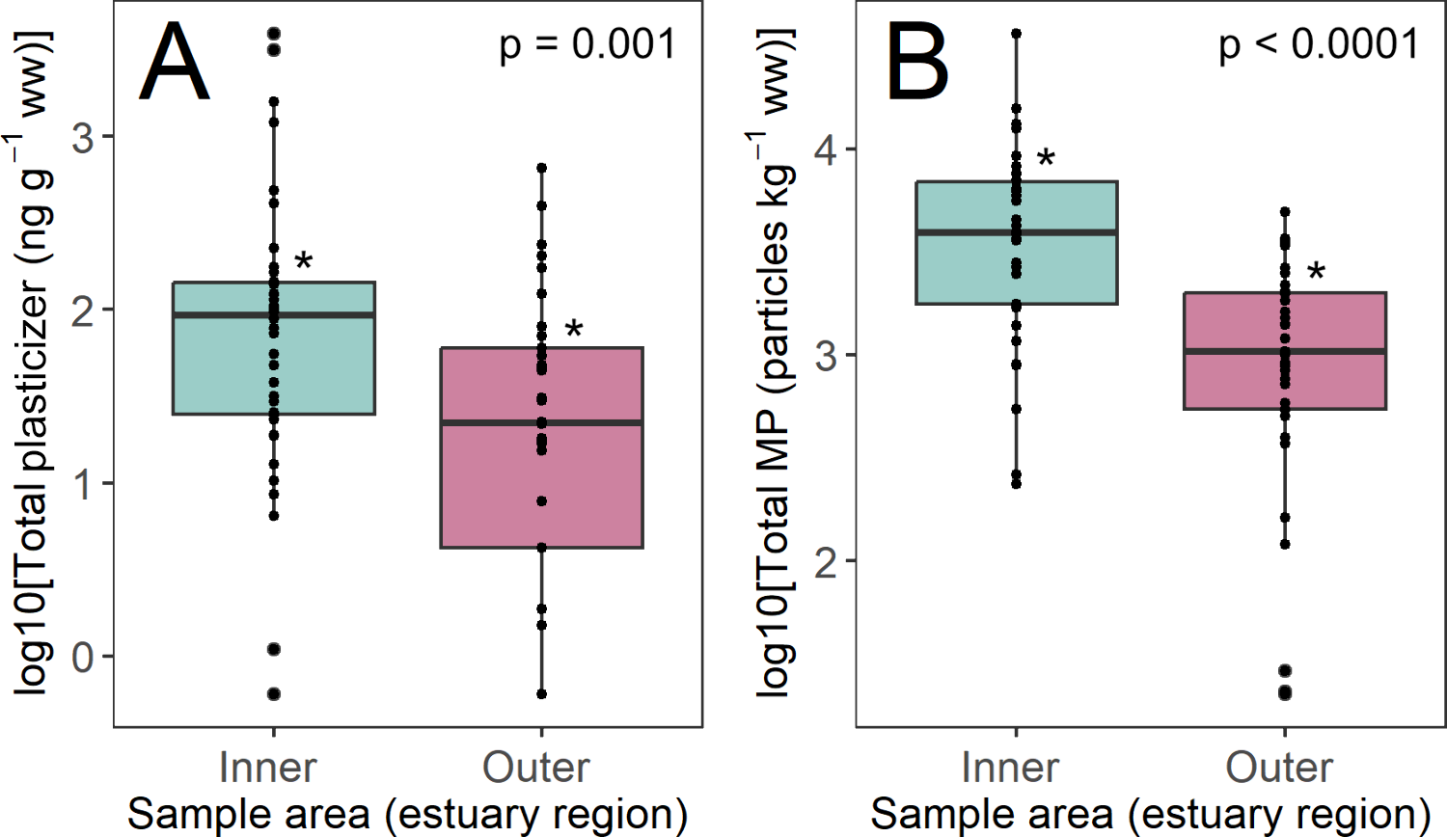
Concentrations of Σplasticizer (ng g^–1^ ww; log10-transformed) (A) and Σmicroplastic (particles kg^–1^ ww; log10-transformed) (B) in the inner and outer Forth estuary; reported *p* values are from LMMs accounting for sampling season (see Section 2.5); differences between sampling areas with *p* values < 0.05 are indicated by an asterisk (*).

The strong evidence for significantly higher levels of microplastics and plasticizers in the inner estuary compared to the outer estuary may be attributed to both flow/sediment dynamics and proximity to sources. Although there remains a lack of systematic studies of the sources of plasticizers in rivers, a number of potential sources have been identified, e.g., unintentional discharges from sites of plastic manufacture,^42^ runoff from sewage sludge-amended agricultural land,^43^ and presence in WWTP effluent.^44^ Subsequent binding of plasticizers to riverine suspended sediments^45^ will consequently result in plasticizer deposition in estuarine bed sediments as increases in salinity as the riverine fresh water enters the estuary result in salting-out of suspended sediments and dissolved contaminants. Furthermore, phthalates themselves are sensitive to changes in salinity; e.g., DEHP had a significantly higher salting constant than other commonly studied environmental contaminants.^46^ As the sorption of plasticizers to sediment appears to be rapid,^46,47^ this may act to confine the transport of plasticizers to upper stages of the estuary (Figure 2).

In addition to riverine inputs, it is also possible that proximity to sources of microplastics and plasticizers may have contributed to the spatial variation in concentrations (Figures 2 and 3, Figures S3 and S4). For instance, the majority of municipal and industrial WWTP outfalls in the Firth of Forth occur upstream of the inner estuary sites (Figure 1), and although the majority of microplastics and plasticizers removed in WWTPs partition to the biosolids and are not released directly into water courses, a fraction does enter through effluent.^44,48^ There is also some evidence to suggest that landfill leachates are potential sources of plasticizers in freshwater and coastal environments,^49,50^ a situation which has been predicted to be exacerbated for other organic contaminants (e.g., PAHs) by increased erosion and flooding of coastal sites.^51,52^ Of the four operational landfill sites within 10 km of the estuary and/or within 5 km of a tributary entering the estuary, three are to the west (upstream) of the inner estuary sites, although none are immediately adjacent to the shoreline or are known to be actively eroding. Thus, while it is possible that WWTPs and landfill sites represent contributors of microplastics and plasticizers to the estuary, source apportionment is challenging given complex estuarine dynamics and competing inputs.

**Figure 3:**
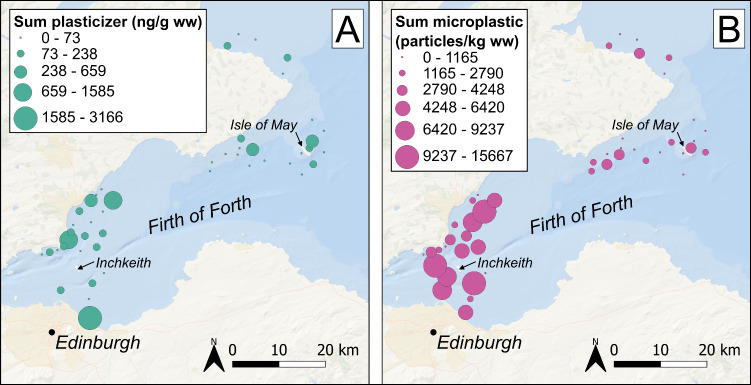
Concentrations of plasticizers (A; ng g^–1^ ww) and microplastics (B; particles kg^–1^ ww) in the Forth estuary in winter 2020–2021 (see Figures S3 and S4 for spring 2021 and summer 2021); nondetects were assigned a value of zero; symbol size classes were determined using Jenks natural breaks optimization.

### 3.2 Plasticizer Profiles in the Firth of Forth Were Dominated by the Legacy Phthalate DEHP

DEHP was the most frequently detected and abundant plasticizer in both the inner (mean 311 ng g^–1^ ww; 70% detection frequency) and outer (63.5 ng g^–1^ ww; 58% DF; Table S2) estuary. This could be expected given that DEHP is the most widely produced plasticizer globally^18^ and longer-chain phthalates have greater affinity for organic matter in sediments.^53^ Hence, DEHP dominated the overall plasticizer profile in the Firth of Forth, accounting for 81–99% of Σplasticizer in each sample area and season (Figure 4), which is consistent with its widespread use and environmental behavior in estuary systems.^54,55^

**Figure 4:**
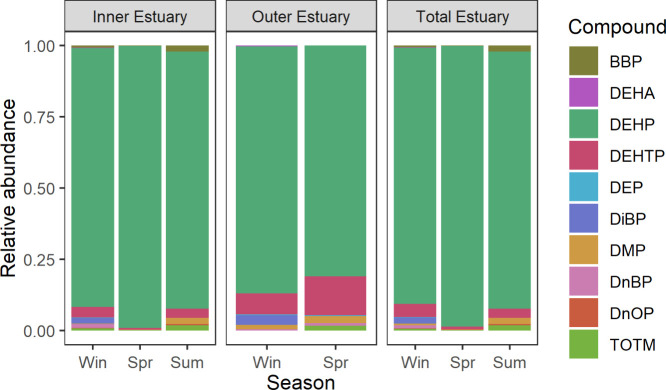
Plasticizer profiles in sediments of the Forth estuary; Win = winter; Spr = spring; Sum = summer; NB: no samples were collected at the outer estuary during summer; therefore, the total estuary concentrations are solely composed of inner estuary samples for that season.

The next most commonly detected plasticizers were DMP, detected in 47% of samples (mean 1.1 ng g^–1^ ww), and the emerging plasticizer DEHTP, found in 41% of samples (mean 7.3 ng g^–1^ ww). DMP is the phthalate with the lowest boiling point and lowest logK_OW_, and thus, it has the potential to be widely transported in the environment relative to other phthalates. DEHTP had the greatest contribution of all of the emerging plasticizers to Σplasticizer in the inner (2.6%), outer (7.8%), and whole estuary (3.4%). DEHTP is a structural isomer of DEHP and exhibits comparable organic matter binding and resistance to degradation.^31^ Terephthalates, such as DEHTP, currently account for ∼15% of the total European plasticizer market and are increasingly used as phthalate replacements.^18^ While mean seasonal DEHTP concentrations at the inner and outer estuary (1.8–12.3 ng g^–1^ ww) were low relative to DEHP, the occurrence of this plasticizer indicates that their increasing use is leading to their presence in estuarine environments.

The medium molecular weight phthalates DnBP, DiBP, and BBP were detected at a wide range of concentrations, although mean concentrations were generally low (Table S2), e.g., 0.3–2.8 ng g^–1^ ww (DnBP), 2.4–3.9 ng g^–1^ ww (DiBP) and nd–21 ng g^–1^ ww (BBP). Detection frequencies of DnBP (15%), DiBP (15%), and BBP (11%) across the entire data set were low. The least abundant phthalates DnOP and DEP had maximum concentrations of 3.1 and 4.5 ng g^–1^ ww and were also infrequently detected (15% and 10%). These results suggest both low and/or localized source intensity and/or relatively low persistence for these phthalates in the estuary.

The emerging plasticizers TOTM and DEHA were also infrequently detected (Table S2) and found at low concentrations; e.g., mean TOTM and DEHA concentrations in the inner estuary were 2.1 ng g^–1^ ww (nd–40.7 ng g^–1^ ww) and 0.3 ng g^–1^ ww (nd–6.4 ng g^–1^ ww). The low concentrations and detection frequency of TOTM in this study are in contrast to previous reports from estuarine and coastal sediments in South Korea, where TOTM has been found to be one of the most abundant emerging plasticizers.^56–59^ This discrepancy may represent differences in regional production and use of phthalate replacements, although there is a lack of detailed regional-scale data concerning the production and usage of different plasticizers, both in time and across different regions, making comparative analysis challenging.

To date, there have been few studies of the occurrence of plasticizers in European estuarine sediments, with most studies focusing on sediments in China and South Korea.^53^ Plasticizer occurrence in systems in South-East Asia will be reflective of a different set of past and current sources than for the Firth of Forth. For example, the relatively low contribution of DnBP and DiBP to Σphthalate in our study is in contrast to sediment studies in China, which typically report absolute mean concentrations of DnBP and DiBP >1 order of magnitude higher than reported here. Correspondingly, these phthalates can account for up to 50% of Σphthalate in Chinese and South Korean estuarine sediments.^17,54,60^ Conversely, mean concentrations of DiBP reported here are comparable to those in studies in South Korea, although previous studies have reported greater DnBP abundance.^56–59^

The least abundant phthalates in the Firth of Forth, based on mean or median concentration, were DMP, DEP, and DnOP (Table S2). DMP was also the second most frequently detected phthalate (46.6% of samples), which indicates widespread occurrence at low concentrations. The low relative abundance of DMP and DEP can be attributed to low production volumes,^18^ more rapid degradation,^31^ and potential that DMP and DEP have lower logK_OW_ values relative to other phthalates (Table S1), meaning they partition less strongly to sediment, allowing them to be transported from the estuary in the tidal flows. The absolute concentrations of DMP, DEP, and DnOP are generally an order of magnitude lower than reported in China^17,54,60^ but similar to those reported in Korea.^56–59^ As there is currently little information about the occurrence of plasticizers in western European estuaries, further work is required to determine whether the results reported here reflect wider estuarine pollution patterns.

### 3.3 Sediments Integrate a Complex Mix of Microplastics That May Arise from a Range of Sources

Before the relative frequency of polymers identified in sediments in the Firth of Forth is interpreted, it is important to consider uncertainty and representativeness of the samples. In turbulent mixed systems where particles are randomly distributed and act independently, the Poisson point process can estimate the sampling error when quantifying microplastics. Estimates of sampling error by the Poisson point process have been demonstrated to be predictive of the measured sampling error when quantifying microplastics in river waters,^61^ and this principle has been applied in the bottom-up evaluation of uncertainty when quantifying microplastics in sediments.^62^ Increasing the number of particles quantified increases the confidence and reduces sampling error when quantifying additional properties, e.g., polymer identity or size distributions. A minimum of 10 particles has been suggested as the requirement for predictions of the sampling error in an individual sample to be accurate.^61,63^ Meanwhile, if 96 microplastic particles are quantified and chemically identified, this information would be associated with a 10% sampling error, while 386 particles require detection for a 5% sampling error.^64^ Others have suggested that, to quantify continuous distributions like particle size, a guideline target of 500 particles should be measured.^65^ When taking individual sediment samples, rarely were such high target numbers reached, and so, we do not estimate sampling error on a sample-by-sample basis. However, our sampling design allows for several relevant groupings of samples to be constructed. The total particle counts associated with each group can be compared against the target guidelines to provide qualitative context on the associated sampling error.

The inner and outer estuary regions represent areas distinguished by proximity to point sources (*n* = 34 and *n* = 36, respectively, for the inner and outer estuary). These areas were sampled across different seasons (winter, spring, and summer); so, the combination of area and season can be considered to be groups (Figure 5). The raw microplastic data quantified between 243 and 1839 individual particles across groups of samples defined by area and season before any correction comparison against LODs. This represents sufficient particle numbers to establish the contribution of different polymers with a sampling error <10% (i.e., >96 microplastics measured), assuming the particles counted were randomly distributed within the mixed sediments. Care was taken to achieve this by mixing sediments immediately prior to subsampling for extraction and analysis. Likewise, when establishing particle size distributions, 2577 and 1767 measured microplastics were used to establish the distribution for the inner and outer estuary, respectively. Note these assessments are qualitative as they are performed on the raw data, while the processed data (>LOD) is used in the final analysis. This sets the context for the uncertainty associated with evaluating polymer profile and size distribution of microplastics in the current assessment.

**Figure 5:**
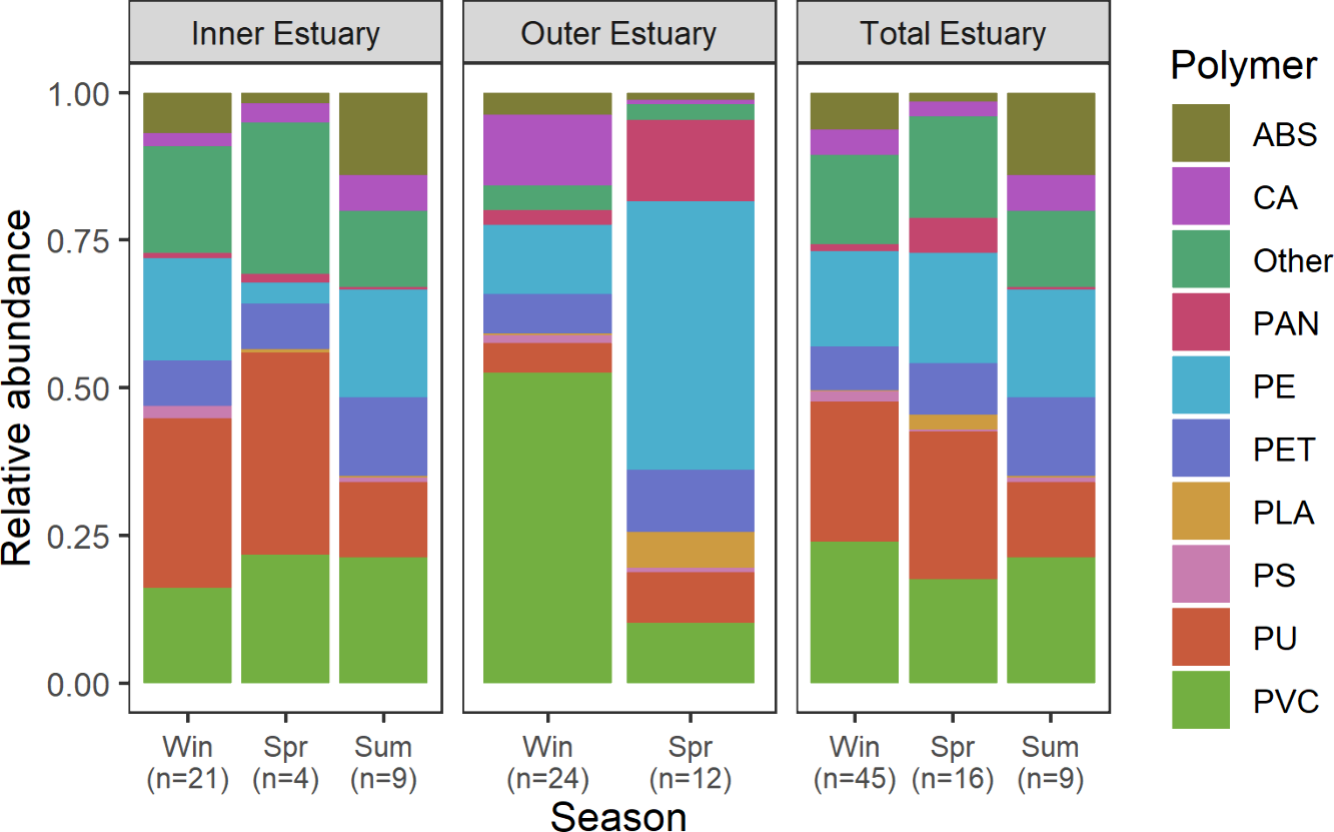
Microplastic profiles (count data; particles kg^–1^ dw; Table S3) in sediments of the Forth estuary; Win = winter; Spr = spring; Sum = summer; NB: no samples were collected at the outer estuary during summer; therefore, the total estuary concentrations are solely composed of inner estuary samples for that season; “Other” includes PA, PMMA, PBT, PPSU, Silicone, EVOH, PSU, POM, PC, PP, EVAc, and PEEK.

Plastics associated with packaging materials that have a density similar to water (e.g., PE and PS) or with paints and foams (e.g., PU) were observed in all sediments, even though in some forms they may be expected to float. This confirms reports of buoyant plastics incorporated into sediments in other environments.^66^ This indicates that heteroaggregation with other particulate matter and freshwater flocs may contribute to sedimentation dynamics of microplastics^67^ and that buoyant polymers may still contribute to sediment exposures to microplastics. The proportion of these buoyant plastics appears higher in the outer estuary compared to the inner; e.g., polymers with densities similar to water (PP, PE, PS, ABS, EVAc) contributed 33% and 22% to total microplastic count in the outer and inner estuary, respectively. This finding is consistent with the hypothesis that floating material, perhaps present as heteroaggregates or fouled microplastics, travels further downstream and is deposited under the higher ionic strengths of the more saline waters of the outer estuary. Likewise, some denser plastics such as PET, which is also largely associated with packaging material, contribute a greater proportion to the total plastics in the inner estuary than in the outer estuary. An exception is the general ubiquity of PVC across both regions. The proportion of microplastic identified as PVC was quite consistent across locations and seasons with the exception of the outer estuary during winter, where around 50% of the microplastic particles quantified were PVC, although these results should be thought of as indicative and not be overinterpreted. Total measured numbers of each polymer are quite low in each individual sample, meaning there is an associated sampling error as understood by the Poisson distribution described earlier. As such, we do not statistically compare the proportion of each polymer in individual samples from the outer and inner estuaries, as any statistical comparison is confounded by this additional source of uncertainty.

It is difficult to quantitatively compare the size distributions of the microplastics found in sediments from the two main sampling areas in the estuary, as the baseline of the blanks cannot be identified on a particle-by-particle basis in the samples and removed from the analysis. However, very few particles >500 μm were observed (8 in total from the inner estuary and 5 in the outer). While there is high confidence that these particles were real contamination in the sediments (no such sized particles were detected in blanks), to establish concentrations of larger particles, e.g., >1000 μm, larger sample volumes than the 15–30 g typically analyzed are required. Thus, comparing microplastic size distribution all the way to 5 mm would require a distinct sample collection and preparation procedure to be developed using high volumes for collection to ensure high numbers of large microplastics for any comparative analysis.

### 3.4 There Was No Evidence for a Correlation between Plasticizer Concentrations and Microplastics (∼25–1000 μm in Diameter) in Sediments of the Firth of Forth

We found no evidence of a relationship between Σmicroplastic (based on either count or mass) and Σplasticizer in sediments at a site level (LMMs of log-10 transformed data; Figure 6; microplastic count: *R* ^2^_LMM_ = 0.002, estimate = −0.07, SE = 0.19, *t* = −0.36, *p* = 0.72; microplastic mass: *R* ^2^_LMM_ = 0.02, estimate = 0.13, SE = 0.10, *t* = 1.2, *p* = 0.23). We also found no evidence of a relationship between PVC number or mass concentrations and Σplasticizer (LMMs of log-10 transformed data; PVC count: *R* ^2^_LMM_ = 0.004, estimate = −0.08, SE = 0.16, *t* = −0.5, *p* = 0.61; PVC mass: *R* ^2^_LMM_ = 0.001, estimate = −0.04, SE = 0.15, *t* = −0.27, *p* = 0.79), despite the fact that >90% of plasticizers are used in PVC^18^ and PVC was detected in 80% of samples and contributed 21% to the mean total microplastic count. The absence of a strong relationship between plasticizers and microplastics on either a mass or a count basis suggests that *in situ* microplastic contamination (∼25–1000 μm) may not be the primary contributor to plasticizer occurrence in sediments of the Forth estuary. This is not to say that plasticizers are not associated with microplastics in this size range, only that other factors not accounted for in the design of this survey also play a role in determining the fate and ultimate distribution of these chemicals in estuarine sediments. These aspects are discussed further in Sections 3.4.1 and 3.4.2.

**Figure 6:**
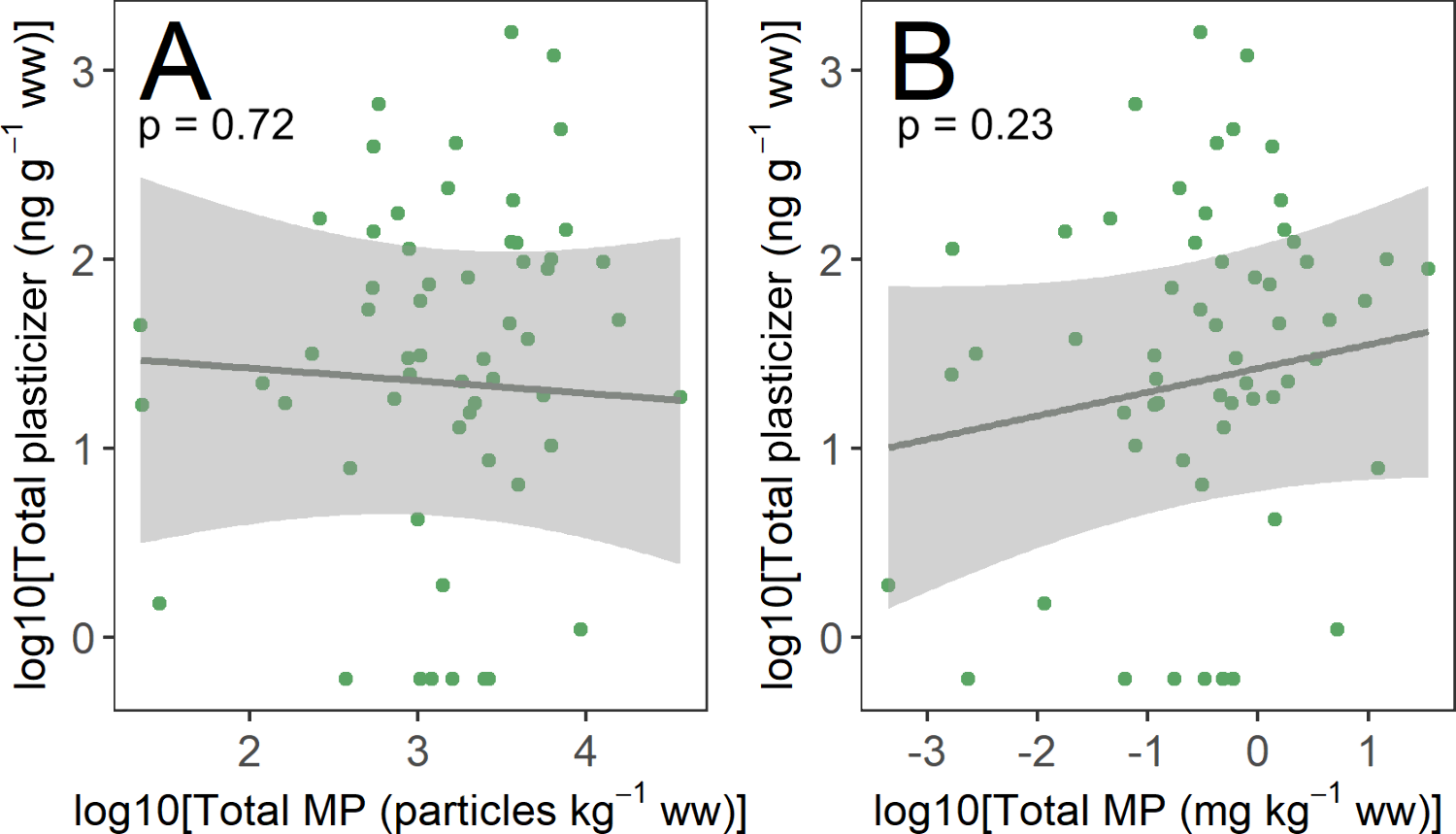
Relationships between Σplasticizer and (A) Σmicroplastic (counts) and (B) Σmicroplastic (mass) in sediments of the Forth estuary; gray lines indicate the predicted values (±95% confidence interval) from linear mixed models; sampling season and area are controlled for in the models (see Section 2.5).

While this is not the first study to investigate the correlation between plasticizers and microplastic concentrations in marine sediments, we believe this to be the most extensive survey of its kind to date and one of the first for estuarine sediments. For instance, previous marine studies have drawn conclusions based on 3 and 5 sampling stations,^68,69^ while here we report on a total of 58 samples from 45 sites. Our study also expands on previous studies by broadening the plasticizers analyzed to include emerging plasticizers which occupied ∼45% of the European market share in 2020, up from ∼12% in 2005.^70^ An emerging picture of similar findings in other environments, including soils, have also reported no definitive link between plasticizers and microplastics of comparable size ranges to those analyzed in our study (≥20–25 μm).^30,71^ The current assessment, through the high spatial resolution in the sampling strategy, brings more confidence in this conclusion of the absence of a clear link between plasticizers and microplastics. There are several contending causes that may explain this absence of a strong correlation.

#### 3.4.1 Might Different Sources and Fates Explain Plasticizer and Microplastic Distribution?

There is likely to be input of both “free” plasticizers (i.e., those not interred within plastic particles) and “bound” plasticizers (i.e., those contained within plastic particles, either from intentional addition or sorption from the surrounding environment) into the rivers which flow into the estuary, e.g., from WWTP and stormwater runoff from urban and agricultural land. In addition, there are a number of potential sources of free plasticizers in close proximity to the estuary itself. These include multiple wastewater discharges, municipal and industrial waste sites, and urban areas (Figure 1).

In order to investigate these potential drivers of the varying distributions of microplastics and plasticizers in the estuary, a principal component analysis of individual contaminant concentrations, in addition to distance from the shoreline and depth in the water column, was carried out. PC1 (16.5%) and PC2 (11.1%) accounted for 27.6% of the variability in the data with the first five principal components accounting for just 49.9% of the variability in the data. The relatively low variability explained by the first two to five principal components highlights the complexity in the mechanisms driving the differential occurrence of plasticizers and microplastics in the Forth estuary and the challenges associated with determining the source-apportionment of these contaminants in estuarine systems. The PCA clearly discriminated between inner estuary sites and outer estuary sites with the 95% confidence intervals for the mean of the groups being distinct from one another (Figure 7A). The sampling areas were separated approximately equally by PC1 and PC2, with the inner estuary being associated positively with both PC1 and PC2, and the outer estuary negatively associated. This primarily reflects that the levels of contaminants were higher at the inner estuary, as discussed in Section 3.4. Similar PCA results were produced when microplastic concentrations were included on a mass basis, although the relative variance explained by the PC1 and PC2 was slightly lower (13.4% and 10.8%; see Figure S5). For consistency with the rest of the results reported in this study, the PCA based on count data will be the focus of the following discussion.

**Figure 7:**
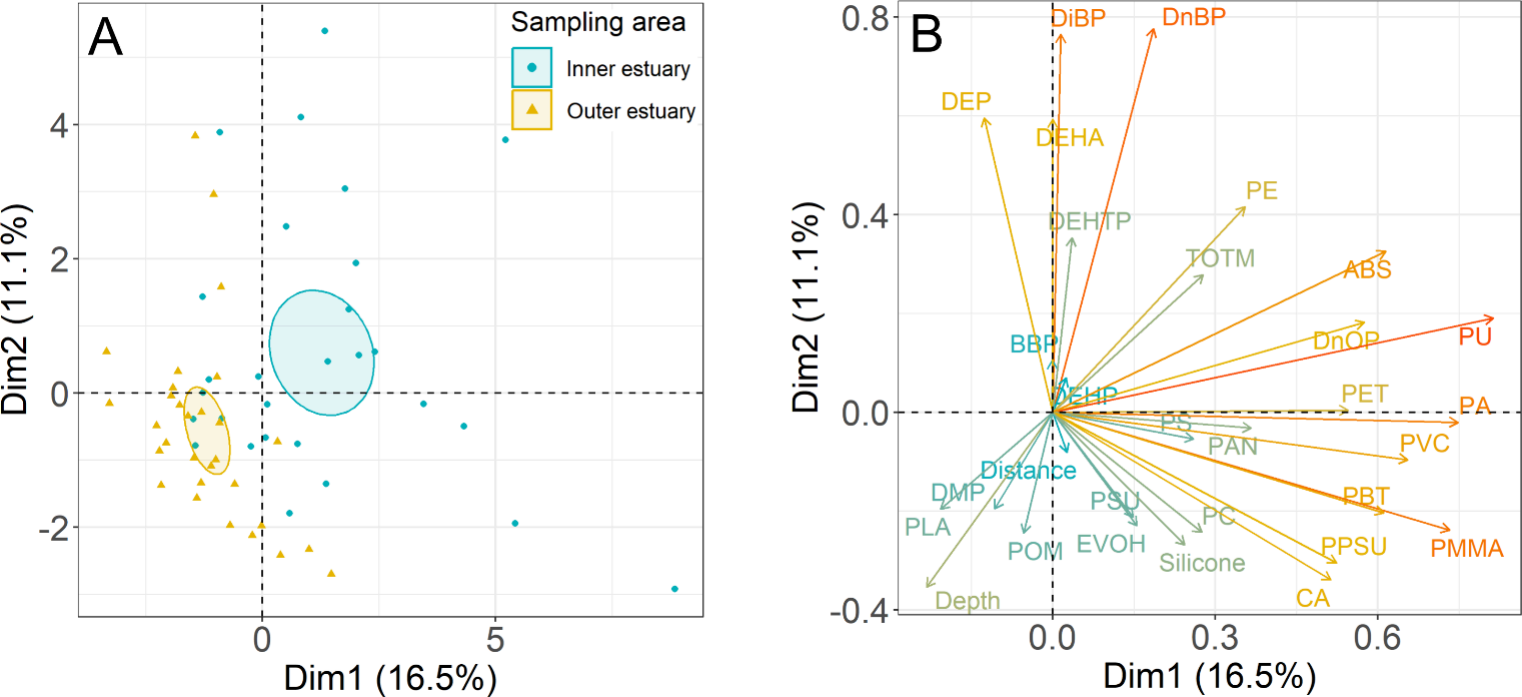
Individual scores (A) and loadings (B) of the first two principal components from a PCA of individual and sum plasticizer and count-based microplastic concentrations, depth in the water column, and distance from the shoreline (see Section 2.5).

The variables with the greatest positive contribution to PC1 included microplastic polymers, e.g., PU (13.6%), PA (11.5%), PMMA (11.1%), PVC (8.8%), and ABS (7.8%). Plasticizers had the greatest positive contribution to PC2, for instance, DnBP (18.4%), DiBP (17.9%), DEP (10.8%), and DEHA (10.7%). The depth of the site in the water column and distance of the site to the shoreline both had a negative association with PC2 (Figure 7B). However, while the depth in the water column also had a negative association with PC1, the distance to the shoreline was almost orthogonal to PC1 (Figure 7B). The results of the PCA indicate that higher levels of many plasticizers were associated with sites that were both shallower and closer to the shore, while a number of individual microplastic polymers were associated with shallower sites but not necessarily associated with decreasing distance from the shoreline. A similar pattern was observed in the mass-based PCA, although the directions of the associations were rotated by ∼90% (Figure S5).

These PCA results provide support to the idea that mechanisms responsible for patterns of plasticizer occurrence in the estuary differ from those that are responsible for those of the microplastics in the size range analyzed (∼25–1000 μm). The negative association of the majority of individual microplastic polymers and plasticizer compounds with depth indicates that these contaminants may primarily enter the estuary from the main tributary (i.e., that a significant source of these contaminants is from the Forth river itself). This is likely related to inputs associated with land uses within the river catchment itself, e.g., runoff from sewage sludge-amended agricultural soils. In the case of some plasticizers (e.g., DiBP and DEHTP), the additional (weaker) negative association with distance from the shoreline (Figure 7B) indicates that sources within the estuary itself may also act as inputs of these plasticizers to the sediments. For instance, there are a number of WWTP outfalls that discharge along the estuary shoreline (Figure 1). While it has been shown that the vast majority of microplastics in the size range analyzed are sequestered into the sewage sludge in UK WWTPs (upward of 99.8%),^28^ the mass balance of plasticizers in sewage works may not be so heavily weighted toward the sludge (although the partitioning of plasticizers to sludge is still dominant).^44^ Therefore, the loading of plasticizers in WWTP effluent may be greater than for microplastics, providing a mechanism that drives differences in the spatial patterns of some of the contaminants (Figure 7B).

In addition to differential sources of microplastics and plasticizers, the processes governing removal of these contaminants in the environment are very different. Biodegradation is the primary removal mechanism for plasticizers in nonaqueous environmental compartments such as sediments,^72^ and although some plasticizers may be relatively long-lasting (e.g., half-lives on the scale of weeks to months),^31,73^ they will eventually degrade. The primary degradation mechanism of (micro)­plastics is likely to be through fragmentation via mechanical and/or photochemical weathering.^74,75^ Half-lives of polymers in marine environments may be on the scale of decades.^76^ Thus, differences in the drivers and time scales of the degradation of plasticizers and microplastics may also act to weaken the relationship between these contaminants.

#### 3.4.2 What Do We Know about Weathering and Fragmentation of Microplastics in This Size Range?

The lack of evidence for a significant measured relationship between these contaminants may also partly result from the size ranges of the particles analyzed. Given the size continuum of plastic waste in the environment spanning several orders of magnitude^77^ and the fact that ∼70–80%^78^ of the marine microplastic burden is estimated to be composed of secondary microplastics, it may be that the microplastic size fraction analyzed in this study had already released its plasticizer content prior to fragmentation and sedimentation. This is consistent with some previous studies of plastic waste and plasticizers in other environments. For example, in the terrestrial environment, correlations between macroplastic litter and plasticizers have been reported, but no clear links between microplastics and plasticizers in soils have been found.^30^

Fragmentation due to weathering typically comprises an initiating event of molecular degradation at the plastic surface and conversion of matrix components to dissolved organic carbon.^2^ The depth of oxidation from weathering such as through UV degradation has limited penetration into the bulk material of plastics. For example, spectral signatures of oxidation are only visible down to a depth of ∼600 μm from the surface of LDPE.^79^ The aging and chemical transformation of the surface of plastic is a prerequisite for the generation of most secondary microplastics through environmental weathering. A rough power law relationship was observed between microplastic particle abundance and decreasing size of the microplastics detected, with the majority in the lowest size category (defined as a width of ≤25 μm) (Figure S2). Very few of the particles detected (7.56%) were ≥100 μm in width. This may explain why there is a poorer correlation between microplastics and plasticizers in sediments than otherwise might be expected. The particles detected may represent microplastics lost from the surface of weathered and disintegrating plastics. We are not aware of any published characterization of the depth profile of organic additives and plasticizers in weathered (micro)­plastics. However, being predominantly <600 μm in size, such fragments may have already lost much of the original additive load either during weathering of the bulk material’s surface prior to fragmentation or due to the higher surface area to volume ratio of particles released allowing rapid leaching. Consistent with this, previous studies have reported almost instantaneous leaching of plasticizers (e.g., DEHP and DEHTP) from microplastics in water^80–82^ and soils^31^ driven by rapid boundary layer diffusion, although subsequent longer-term release rates of these plasticizers (e.g., over weeks-months) appear slower with the majority of plasticizer released over extended time scales.^31,80,81^

Simulation studies or direct isolation and measurement of environmentally weathered microplastics would be required to confirm whether these small microplastics generated via weathering of larger plastic items contain a significant load of plasticizers. Larger plastic litter can be collected from, e.g., beach shorelines, and plasticizers and other organic pollutants can be quantified.^83,84^ However, for smaller microplastics (<1 mm), which will be available to a greater range of organisms, such investigations have not yet been conducted. The separation techniques used to isolate these microplastics from environmental samples, such as those followed in the current study, may not be suitable for plasticizer content assessment. This is due to the fact that these techniques: (a) do not completely isolate microplastics from interfering sediment material, leaving a residual matrix that may contain plasticizers; (b) require chemical digestions which may chemically transform the plasticizers in the microplastics. As such, to the best of our knowledge, this question remains unanswered.

#### 3.4.3 Are Smaller Secondary Microplastics (<1 mm) a Significant Vector for Transport of Plastic Additives?

While there is discussion around the relevance of microplastics as a vector for exposure to adsorbed pollutants in relation to other suspended particulate matter which may outnumber microplastics by many orders of magnitude,^85^ the unique application of plasticizers in the synthesis of plastics gives reason to consider the role of microplastics (and larger plastic items) in determining overall exposure to these chemicals in wildlife and through food chains.^86^ The potential for differential fate of free plasticizers as compared to those still within the plastic material, which may act as a harbor for the persistence and wider distribution of these additives than in the unbound state, also raises a valid concern to be investigated.

To conclude whether microplastics in this size range contribute to the transfer of plastic additives and their internal exposure and accumulation in wildlife, studies utilizing a combination of orthogonal analytical techniques, capable of measuring the total mass of individual polymers in environmental samples irrespective of particle size (e.g., pyrolysis-GC-MS) in conjunction with count-based methods (such as the imaging μ-FTIR used in our study), are warranted. Sample preparation procedures that can isolate microplastics in a way that their additive content maintains integrity to its environmental state and upon which chemical analyses for plasticizers can be performed are also required.

### 3.5 The Presence of Plasticizers and Microplastics Represents an Unknown Exposure Risk in the Forth Estuary

The uptake of phthalates and emerging plasticizers has been demonstrated in a range of marine taxa, e.g., fish,^87–89^ molluscs,^87,88,90^ and crustaceans.^87,89^ Additionally, ingestion of microplastics has been demonstrated in ∼1500 species globally,^5^ with a number of mechanisms of toxicity postulated (e.g., impacts arising from physical interactions with microplastics, oxidative stress, and neurotoxicity).^91^ For some proposed mechanisms of toxicity, ingestion of microplastics is a required precursor to adverse effects, such as through the food dilution mechanism.^92^ The Firth of Forth is home to a number of benthic invertebrate and fish species that may be particularly exposed to plasticizer and microplastic contamination due to feeding patterns which can lead to direct exposure through ingestion of sediment.^93,94^ Several previous assessments from other study locations indicate the possibility of local hotspots of microplastic exposure that may already exceed effect thresholds. These hotspots typically form in waters heavily polluted with buoyant particles or in enclosed coastal regions (e.g., the San Francisco Bay area)^95^ but also potentially a small number in UK freshwater and estuarine environments.^96^

The phthalate DEHP has been associated with endocrine disruption in medaka and salmon,^97,98^ immunotoxicity in trout,^99^ and cytotoxicity and genotoxicity in sea bass.^10^ DEHP may also harm development in marine copepods.^100^ Therefore, the occurrence of some phthalates with known toxicity (e.g., DEHP) in estuary sediments with concentrations of almost 4000 ng g^–1^ ww indicates that plasticizers present an exposure of uncertain risk to some species and potentially contribute to the total load of contaminants in biota in the Forth estuary. However, it is difficult to link such results of sediment concentrations that lead to exposure over highly extended time scales to the effect levels seen in the majority of toxicity studies, which are based on experiments that administer the chemical through diet conducted via in vitro tests. Although we found that emerging plasticizers represent a small proportion of total plasticizer concentrations, the use of emerging plasticizers continues to increase as phthalates are phased out.^18^ To date, there are only a handful of studies of the occurrence of emerging plasticizers in marine species. Despite relatively low abundance, some emerging plasticizers have been detected at similar levels to phthalates in amphipods,^101^ fish, and seagrass,^102^ although in the absence of extensive ecotoxicity data, their risks remain largely unknown.^53^

The Firth of Forth is recognized as one of the most important estuaries in the UK for its wildlife and conservation value. The island of Inchkeith (inner estuary) and the Isle of May (outer estuary) host nationally important colonies of protected seabirds, such as shags, common guillemots, Atlantic puffins, and razorbills.^103^ Furthermore, Bass Rock (outer estuary) is an internationally important breeding ground for the northern gannet, representing ∼13% of the global breeding population of the species.^104^ Sandeels and benthic fish are primary constituents of the diets of these seabirds.^103^ Thus, due to feeding behaviors and the occurrence of plasticizers and microplastics in the estuary sediments, there is potential for the bioaccumulation of these contaminants into sandeels and other prey fish and ultimately into seabird species in the Firth of Forth. To date, the exposure, accumulation, and potential for effects of plasticizers and microplastics in seabirds in the Firth of Forth have not been assessed. However, correlations between large plastic items and microplastic presence in tissues with pathologically significant tissue and cellular level sublethal effects are being reported in other wild seabird populations.^105^ The concentrations of plasticizers and microplastics in the sediments measured here likely indicate a relatively low, but likely year-round exposure potential and risk for wildlife in and around the estuary.

## Supplementary Material



## Notes

Additional information, data, and analyses to complement the findings discussed in the main text: information regarding the plasticizers analyzed in this study (Table S1); concentrations of the plasticizers and microplastics in sediments of the Firth of Forth (Table S2 and Table S3); quality control parameters (blanks and spiked recoveries) for microplastics analysis (Figure S1); size distributions of microplastics in sediments and blank samples (Figure S2); concentrations of plasticizers and microplastics in sediments of the Firth of Forth in spring and summer (Figure S3 and Figure S4); scores and loadings of the first two principal components from a PCA of plasticizer and microplastic concentrations (mass-based microplastic concentrations), water column depth, and distance from the shoreline (Figure S5); information regarding microplastic size class definitions (Appendix S1); contamination controls for microplastic and plasticizer analysis (Appendix S2) (PDF)
