# Supplementary material for: Are Microplastic (∼25–1000 μm) and Plasticizer Concentrations Correlated in Sediments of an Urbanized UK Estuary?
Source: Environ Sci Technol. 2025 Dec 4;59(49):26781–95. doi: 10.1021/acs.est.5c12721 (PMC12713724; doi:10.1021/acs.est.5c12721)
Supplement: Supplementary file 1 [file es5c12721_si_001.pdf]

Supporting Information for:

Are microplastic (~25-1000 µm) and plasticiser concentrations correlated in sediments of an urbanised UK estuary?

Alex Billings[AB], Richard K. Cross[C], Francis Daunt[D], Justyna P. Olszewska[D], Amy Pickard[D], Maria I. Bogdanova[D], Ruairidh Cox[C], Kevin C. Jones[B], David J. Spurgeon[C], M. Glória Pereira[A]*

[A] *UK Centre for Ecology & Hydrology, Library Avenue, Bailrigg, Lancaster, LA1 4AP, UK*

[B] *Lancaster Environment Centre, Lancaster University, Lancaster, LA1 4YQ, UK*

[C] *UK Centre for Ecology & Hydrology, Benson Lane, Crowmarsh Gifford, Wallingford, OX10 8BB, UK*

[D] *UK Centre for Ecology & Hydrology, Bush Estate, Penicuik, EH26 0QB, UK*

[*] *Corresponding author, email: alebil@ceh.ac.uk*

Tables

Table S1: The 7 phthalate and 3 emerging plasticisers analysed in this study 1

Table S2: Plasticiser concentrations in sediments of the Firth of Forth 2

Table S3: Microplastic concentrations in sediments of the Firth of Forth 3

Figures

Figure S1: A) summary of the background counts in blanks distinguished by polymer used in the calculation of limits of detection (n=6). B) Summary of the percentage recovery of polystyrene (PS) and polyamide (PA) representative materials spiked into water 4

Figure S2: Size distribution of all microplastics measured in the inner estuary near Inchkeith (A) the outer estuary around the Isle of May (B), the total combined size distribution in all sediment samples (C) and in the blank samples (D) 5

Figure S3: Concentrations of plasticisers (A) and microplastics (B) in the Forth estuary in spring 2021 6

Figure S4: Concentrations of plasticisers (A) and microplastics (B) in the Forth estuary in summer 2021 7

Figure S5: Individual scores (A) and loadings (B) of the first two principal components from a PCA of individual plasticiser and microplastic concentrations (with mass-based microplastic concentrations), depth in the water column, and distance from the shoreline 8

Appendices

Appendix S1: Information regarding lowest pixel size and resulting definition of lowest microplastic size class 9

Appendix S2: Contamination controls for microplastics and plasticiser analyses 10

References 12

Table S1: The 7 phthalate and 3 emerging plasticisers analysed in this study; solubility in water is at 25 °C unless otherwise stated; internal standards and recovery standards refer to the deuterated-labelled compounds used in the quantification and analysis of each plasticiser compound.

Name and acronym		Formula	Log K_{OW}	S_{H2O} (mg L^{-1})	CAS	Internal standard	Recovery standard
Dimethyl phthalate	DMP	$C_{10}H_{10}O_4$	1.61 [A]	4000 [B]	131-11-3	DEP-d4	DnBP-d4
Diethyl phthalate	DEP	$C_{12}H_{14}O_4$	2.54 [A]	1080 [B]	84-66-2	DEP-d4	DnBP-d4
Di-iso-butyl phthalate	DiBP	$C_{16}H_{22}O_4$	4.27 [A]	6.2 (24 °C) [B]	84-69-5	DEP-d4	DnBP-d4
Di-n-butyl phthalate	DnBP	$C_{16}H_{22}O_4$	4.27 [A]	11.2 [B]	84-74-2	DEP-d4	DnBP-d4
Benzyl butyl phthalate	BBP	$C_{19}H_{20}O_4$	4.7 [A]	2.69 [B]	85-68-7	DEHP-d4	DnOP-d4
Diethylhexyl phthalate	DEHP	$C_{24}H_{38}O_4$	7.73 [A]	0.27 [B]	117-81-7	DEHP-d4	DnOP-d4
Di-n-octyl phthalate	DnOP	$C_{24}H_{38}O_4$	7.73 [A]	0.022 [B]	117-84-0	DEHP-d4	DnOP-d4
Diethylhexyl adipate	DEHA	$C_{22}H_{42}O_4$	6.83 [C]	0.78 (22 °C) [B]	103-23-1	DEHP-d4	DnOP-d4
Diethylhexyl terephthalate	DEHTP	$C_{24}H_{38}O_4$	8.39 [D]	4.0 (20 °C) [B]	6422-86-2	DEHP-d4	DnOP-d4
Trioctyl trimellitate	TOTM	$C_{33}H_{54}O_6$	9.3 [E]	3.9 x 10^{-4} [B]	3319-31-1	DEHP-d4	DnOP-d4

[A](Net et al., 2015); [B]PubChem online database; [C](US CPSC, 2018); [D](ANSES, 2015); [E](ECCC, 2018).

Table S2: Plasticiser concentrations in sediments of the Firth of Forth (ng g^{-1} ww); samples <LOD were assigned a value of zero so as to avoid overestimation, and included in the calculation of the mean and median; RA% = relative abundance; DF% = detection frequency; nd = not detected.

		DMP	DEP	DiBP	DnBP	BBP	DEHP	DnOP	Σphthalate	DEHA	DEHTP	TOTM	Σemerging	Σplasticiser
**Whole estuary (n=73)**	**Mean**	1.1	0.2	3.2	1.7	1.1	199.1	0.3	206.6	0.2	7.3	1.2	8.7	215.3
	**Median**	nd	nd	nd	nd	nd	29.1	nd	40.1	nd	nd	nd	2.6	45.6
	**RA%**	0.5	0.1	1.5	0.8	0.5	92.5	0.1	95.9	0.1	3.4	0.6	4.1	100.0
	**Range**	nd-10.3	nd-4.5	nd-49.8	nd-82.1	nd-26.4	nd-3932.8	nd-3.1	nd-3932.8	nd-6.4	nd-94.1	nd-40.7	nd-100.6	nd-3932.8
	**DF%**	46.6	11.0	15.1	15.1	11.0	64.4	15.1	90.4	17.8	41.1	20.5	57.5	90.4
**Inner estuary (n=40)**	**Mean**	1.0	0.2	3.9	2.8	2.1	311.0	0.5	321.3	0.3	8.7	2.1	11.0	332.4
	**Median**	nd	nd	nd	nd	nd	47.4	nd	69.8	nd	nd	nd	3.4	92.6
	**RA%**	0.3	0.1	1.2	0.8	0.6	93.6	0.1	96.7	0.1	2.6	0.6	3.3	100.0
	**Range**	nd-10.3	nd-4.5	nd-49.8	nd-82.1	nd-26.4	nd-3932.8	nd-3.1	nd-3932.8	nd-6.4	nd-94.1	nd-40.7	nd-100.6	0-3932.8
	**DF%**	42.5	5.0	15.0	17.5	20.0	70.0	27.5	97.5	17.5	37.5	32.5	62.5	97.5
**Outer estuary (n=33)**	**Mean**	1.2	0.2	2.4	0.3	nd	63.5	nd	67.5	0.2	5.7	0.1	6.0	73.5
	**Median**	0.4	nd	nd	nd	nd	11.7	nd	17.1	nd	nd	nd	1.0	22.2
	**RA%**	1.6	0.3	3.2	0.4	nd	86.4	nd	91.9	0.2	7.8	0.2	8.1	100.0
	**Range**	nd-5.9	nd-3.6	nd-48.6	nd-4.5	nd	nd-645.5	nd	nd-659.3	nd-1.2	d-24.2	nd-2.2	nd-24.2	0-659.3
	**DF%**	51.5	18.2	15.2	12.1	nd	57.6	nd	81.8	18.2	45.5	6.1	51.5	81.8

Table S3: Microplastic concentrations in sediments of the Firth of Forth (particles kg^{-1} dw); samples <LOD were assigned a value of zero so as to avoid overestimation, and included in the calculation of the mean and median; RA% = relative abundance; DF% = detection frequency; nd = not detected.

		PP	PE	PVC	PU	PET	PS	ABS	PA	PC	PMMA	POM	CA	EVAc	EVOH	PAN	PBT	PEEK	PPSU	PSU	silicone	PLA	∑MP
**Whole estuary (n=70)**	**Mean**	nd	1351	1664	1712	702	88	481	499	3	500	9	317	nd	15	204	90	nd	44	22	14	73	7817
	**Median**	nd	684	641	nd	263	nd	nd	nd	nd	nd	nd	nd	nd	nd	nd	nd	nd	nd	nd	nd	nd	4564
	**RA%**	nd	17.3	21.3	21.9	9.0	1.1	6.2	6.4	0.0	6.4	0.1	4.0	nd	0.2	2.6	1.2	nd	0.6	0.3	0.2	0.9	100.0
	**Range**	nd	0-9161	0-23892	0-39231	0-8282	0-2142	0-3809	0-10309	0-156	0-13413	0-308	0-2554	nd	0-436	0-8050	0-2793	nd	0-2810	0-732	0-693	0-2927	0-106544
	**DF%**	nd	55.7	80.0	44.3	54.3	22.9	45.7	12.9	4.3	10.0	4.3	32.9	nd	4.3	28.6	5.7	nd	5.7	4.3	5.7	17.1	95.7
**Inner estuary (n=34)**	**Mean**	nd	1728	2364	3280	1136	146	904	997	5	1029	nd	443	nd	13	117	186	nd	85	24	6	29	12547
	**Median**	nd	1584	1066	1065	379	nd	520	nd	nd	nd	nd	nd	nd	nd	nd	nd	nd	nd	nd	nd	nd	8619
	**RA%**	nd	13.8	18.8	26.1	9.1	1.2	7.2	7.9	0.0	8.2	nd	3.5	nd	0.1	0.9	1.5	nd	0.7	0.2	0.0	0.2	100.0
	**Range**	nd	0-6723	0-23892	0-39231	0-8282	0-2142	0-3809	0-10309	0-156	0-13413	nd	0-2554	nd	0-436	0-1148	0-2793	nd	0-2810	0-441	0-156	0-489	341-106544
	**DF%**	nd	70.6	88.2	70.6	67.6	29.4	70.6	23.5	2.9	20.6	nd	38.2	nd	2.9	35.3	11.8	nd	5.9	5.9	5.9	11.8	100.0
**Outer estuary (n=36)**	**Mean**	nd	996	1003	231	293	34	82	29	2	nd	18	197	nd	17	287	nd	nd	6	20	22	115	3350
	**Median**	nd	nd	422	nd	nd	nd	nd	nd	nd	nd	nd	nd	nd	nd	nd	nd	nd	nd	nd	nd	nd	2242
	**RA%**	nd	29.7	29.9	6.9	8.7	1.0	2.4	0.9	0.1	nd	0.5	5.9	nd	0.5	8.6	nd	nd	0.2	0.6	0.7	3.4	100.0
	**Range**	nd	0-9161	0-7601	0-4185	0-2124	0-308	0-973	0-1055	0-44	nd	0-308	0-1712	nd	0-323	0-8050	nd	nd	0-133	0-732	0-693	0-2927	0-22713
	**DF%**	nd	41.7	72.2	19.4	41.7	16.7	22.2	2.8	5.6	nd	8.3	27.8	nd	5.6	22.2	nd	nd	5.6	2.8	5.6	22.2	91.7

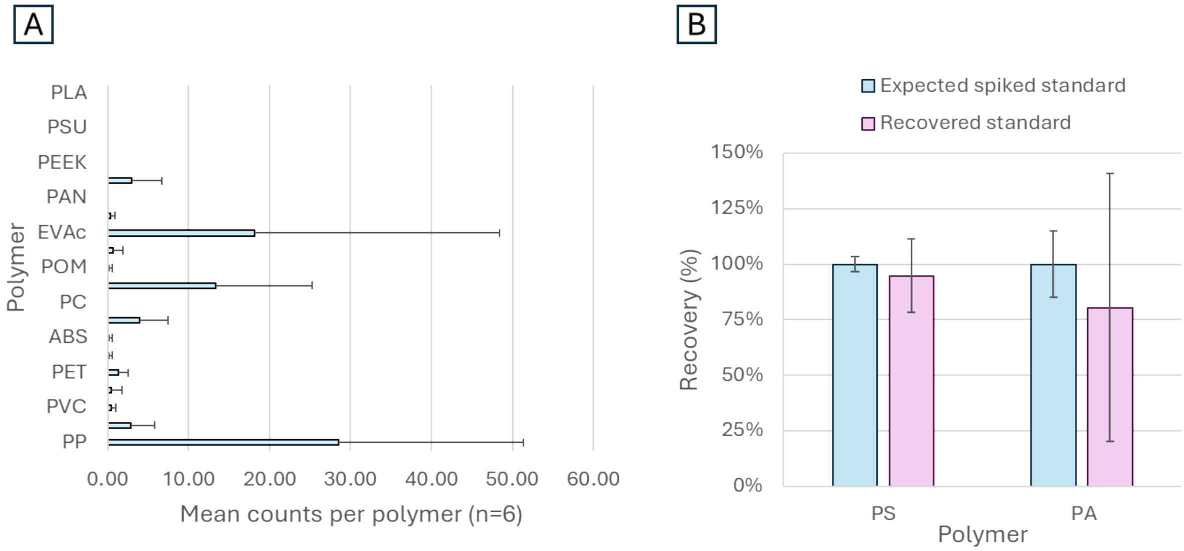

Figure S1: A) summary of the background counts in blanks distinguished by polymer used in the calculation of limits of detection (n=6). B) Summary of the percentage recovery of polystyrene (PS) and polyamide (PA) representative materials spiked into water and followed through the sample preparation procedure alongside sample batches to indicate recovery (n=5). Error bars in (B) represent the standard deviation either in the original stock preparation of the spike standard, or in the recovered standard from the sample preparation procedure.

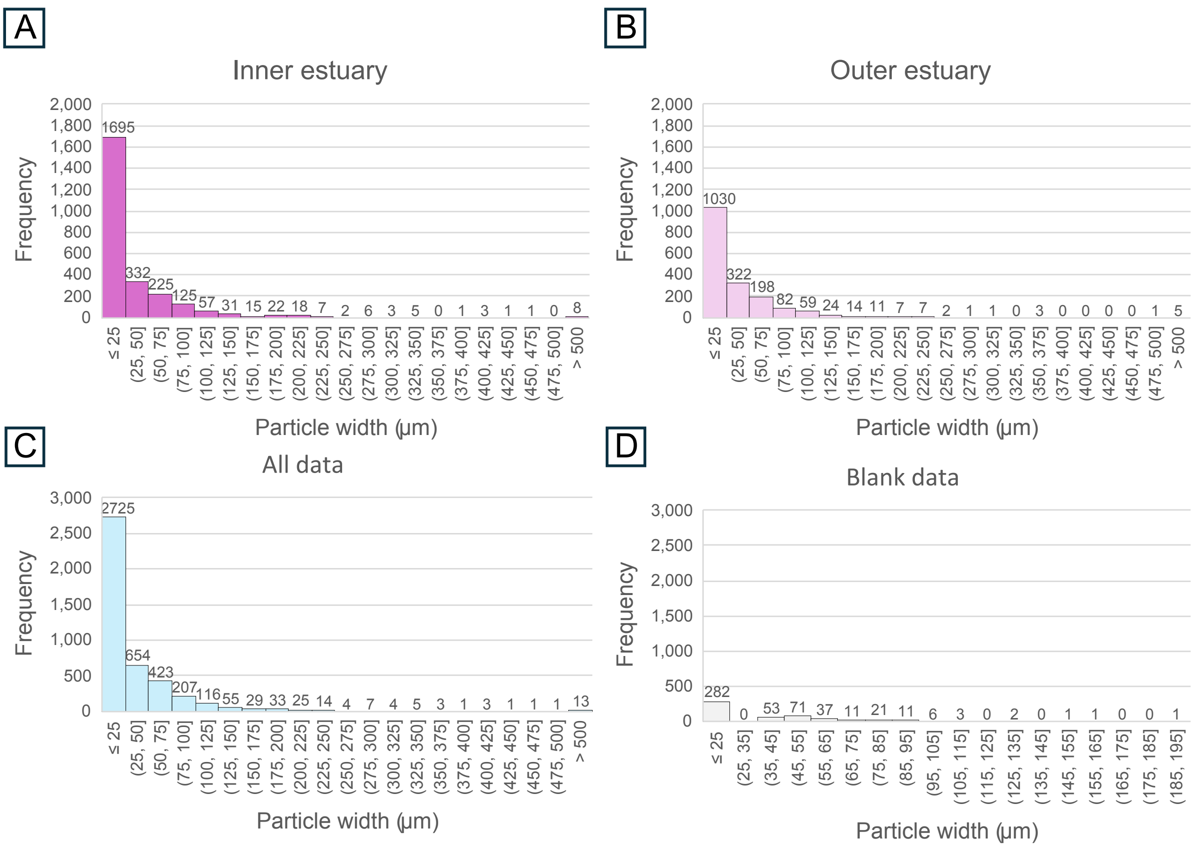

Figure S2: Size distribution of all microplastics measured in the inner estuary near Inchkeith (A, # sites = 34) the outer estuary around the Isle of May (B, # sites = 36), the total combined size distribution in all sediment samples (C) and in the blank samples (D).

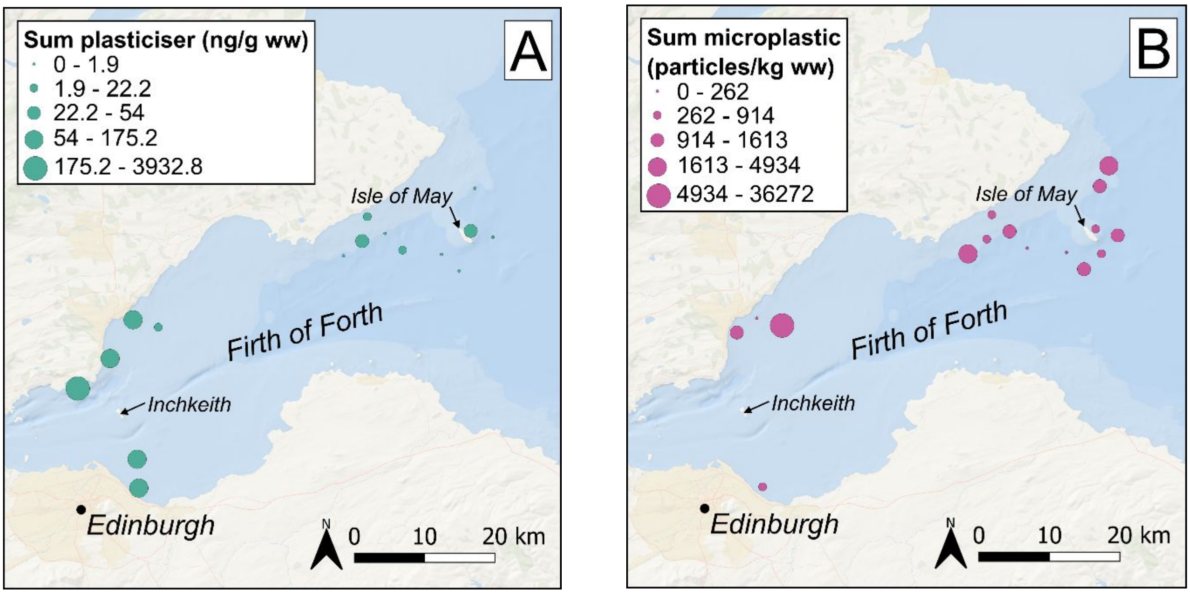

Figure S3: Concentrations of plasticisers (A; ng g^{-1} ww) and microplastics (B; particles kg^{-1} ww) in the Forth estuary in spring 2021; non-detects were assigned a value of zero; symbol size classes were determined using Jenks natural breaks optimisation.

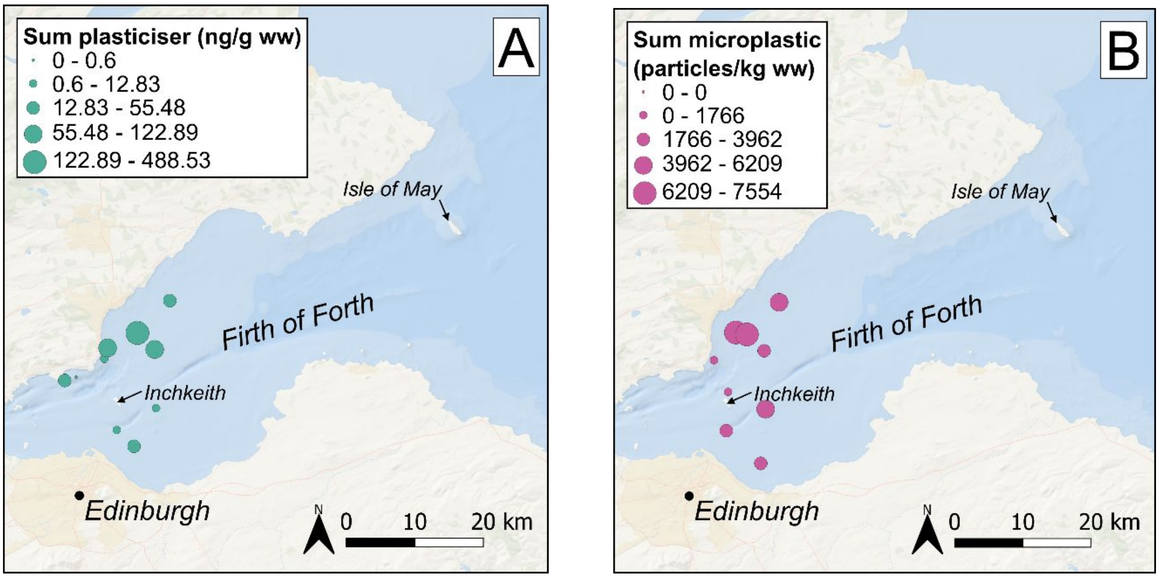

Figure S4: Concentrations of plasticisers (A; ng g^{-1} ww) and microplastics (B; particles kg^{-1} ww) in the Forth estuary in summer 2021; non-detects were assigned a value of zero; symbol size classes were determined using Jenks natural breaks optimisation.

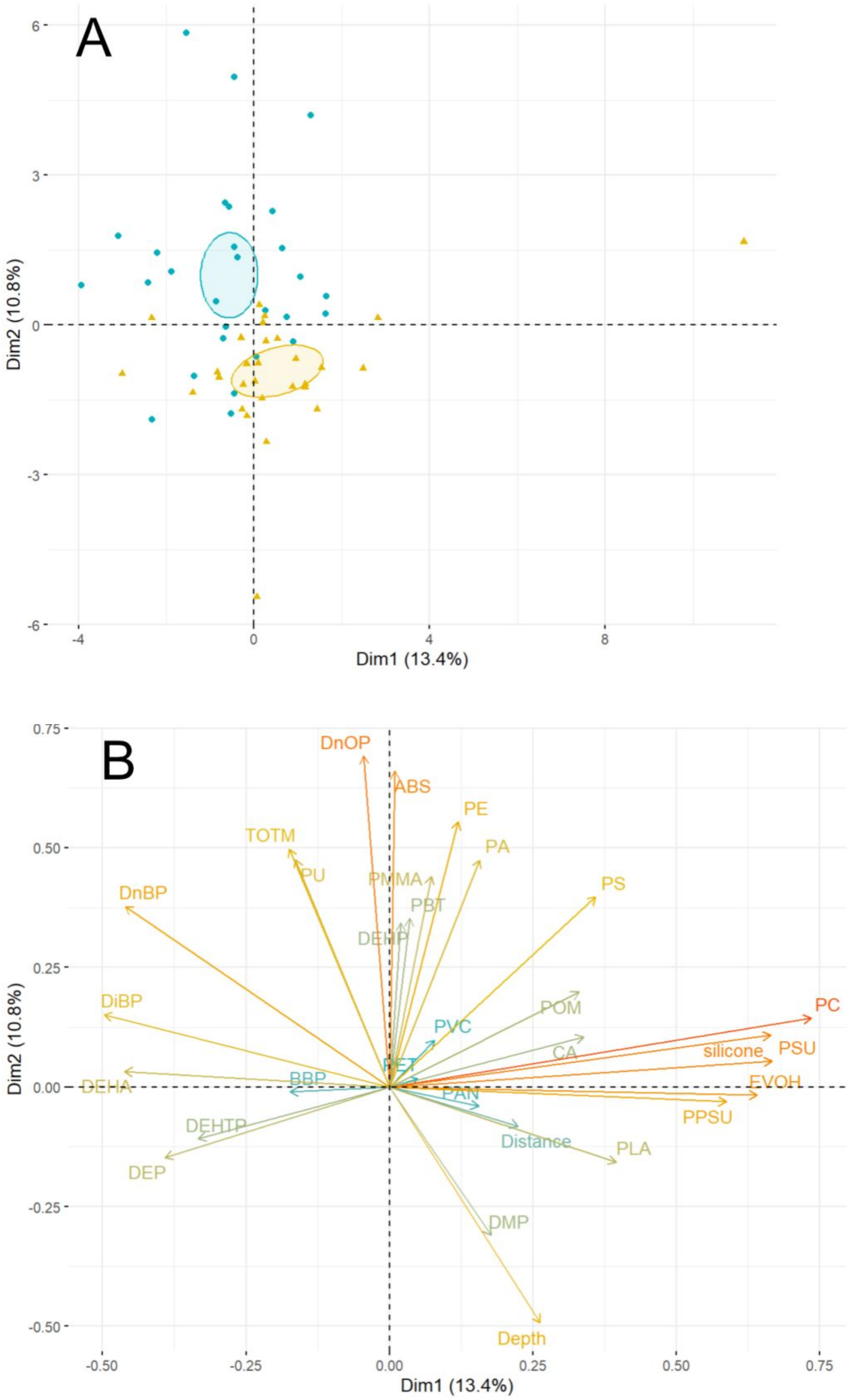

Figure S5: Individual scores (A) and loadings (B) of the first two principal components from a PCA of individual plasticiser and microplastic concentrations (with mass-based microplastic concentrations), depth in the water column, and distance from the shoreline.

Appendix S1: Information regarding lowest pixel size and resulting definition of lowest microplastic size class.

The lower category of size is defined as ≥25 μm as this is the pixel size resolution of the FTIR scans. It is technically possible that particles smaller than 25 μm may still generate sufficient signal to be picked up in the analysis as a single pixel, and so be characterised with a width of 25 μm. However, it is not possible to be quantitative as to the proportion of microplastics smaller than 25 μm in this category without use of an orthogonal analytical technique that can measure particles to a smaller size resolution.

Appendix S2:Contamination controls for microplastics and plasticiser analyses.

For microplastics, measures included wearing cotton lab-coats during sample preparation and analysis, only opening samples to the air in a biological safety cabinet under HEPA filtered air (removing 99.999% of particles >0.3 μm in size), minimising any plastic materials from contact with the samples, cleaning all glassware before use with filtered water and filtering all reagents prior to use (in both cases using 1.2 μm glass fibre filters). Full procedural blanks were run alongside each batch of samples in accordance with the methods previously employed to establish limits of detection for μ-FTIR analysis of microplastics in complex environmental samples (Horton et al., 2021; Johnson et al., 2020).

Contamination control measures used during microplastics sampling, processing and analysis:

**Contamination control method**	**Description**
Material substitution: Limiting plastic in equipment	Where possible, non-plastic or uncommon plastic substitutes were used during sample preparation, including natural fibre brushes, glass Pasteur pipettes, stainless steel buckets, stainless-steel or aluminium filter rigs, stainless steel or pure silver filters, FEP/ETFE wash bottles and glass bottles with PTFE lined lids and ETFE pouring rings for all sampling and processing vessels. All equipment was washed with filtered (<0.7 μm) RO water prior to use.
Clean air conditions: Limiting airborne contamination	HEPA filter removes 99.999% of particles >0.3 μm in size. All processing steps in the laboratory were performed under these or equivalent conditions when possible. When outside of the safety cabinet, all equipment/samples was covered with clean aluminium foil. If equivalent conditions could not be met, this was documented. A systematic use of procedural blanks allowed all data to be corrected for any unavoidable background contamination.
Reagent filtration: Limiting contamination from reagents	All reagents used in microplastic processing were filtered through a glass fibre filter with a pore size smaller than the lower limit of detection for the analysis (e.g. 1.2 μm) to remove any particulates prior to use. A systematic use of procedural blanks allowed all data to be corrected for any unavoidable background contamination.
Cotton lab coats: Limiting cross contamination from synthetic clothing	All laboratory processing was performed by operators wearing 100% cotton lab-coats.
Demonstrate negligible equipment carry over:	Stainless steel filters were commonly used to concentrate samples. Similarly glass beakers and filtering equipment were reused between samples. All stainless-steel disc filters were sonicated and washed between samples with detergent, RO or DI water and finally filtered (<0.7 μm) water. Other equipment followed the clean

Limiting sample cross contamination	washing procedure below. Absence of carry over between samples should be demonstrated if equipment is to be re-used.
Clean washing procedure: Limiting contamination during equipment preparation	All equipment and glassware was washed using only natural fibre scouring brushes to prevent contamination during washing and rinsed repeatedly with filtered RO or DI water before air drying under foil to prevent airborne contamination

Laboratory controls used during the extraction and analysis of plasticisers from the sediments were based on those used in a previous study.(Billings et al., 2023) Laboratory glassware was soaked overnight in Decon 90 and heated in a muffle furnace at 450 °C for a minimum of 2 hours prior to use (non-volumetric glassware only)(Fankhauser-Noti and Grob, 2007) to reduce the contamination of phthalates. Glassware was also rinsed 2 x with DCM immediately prior to use. Operators wore cotton lab coats, and solid reagents were heated in a muffle furnace at 450 °C overnight. The use of plasticware was avoided, except for the PTFE lids of the microwave extraction vessels and gas chromatography sample vials. As discussed above, multiple procedural blanks were carried out and any contamination was accounted for in data processing.

References

ANSES, 2015. Risk Management Options Analysis (DEHTP).

Billings, A., Carter, H., Cross, R.K., Jones, K.C., Pereira, M.G., Spurgeon, D.J., 2023. Co-occurrence of macroplastics, microplastics, and legacy and emerging plasticisers in UK soils. Science of The Total Environment 880, 163258. https://doi.org/10.1016/j.scitotenv.2023.163258

ECCC, 2018. Draft screening assessment: Trimellitates group.

Fankhauser-Noti, A., Grob, K., 2007. Blank problems in trace analysis of diethylhexyl and dibutyl phthalate: Investigation of the sources, tips and tricks. Analytica Chimica Acta 582, 353–360. https://doi.org/10.1016/j.aca.2006.09.012

Horton, A.A., Cross, R.K., Read, D.S., Jürgens, M.D., Ball, H.L., Svendsen, C., Vollertsen, J., Johnson, A.C., 2021. Semi-automated analysis of microplastics in complex wastewater samples. Environmental Pollution 268, 115841. https://doi.org/10.1016/j.envpol.2020.115841

Johnson, A.C., Ball, H., Cross, R., Horton, A.A., Jürgens, M.D., Read, D.S., Vollertsen, J., Svendsen, C., 2020. Identification and Quantification of Microplastics in Potable Water and Their Sources within Water Treatment Works in England and Wales. Environ. Sci. Technol. 54, 12326–12334. https://doi.org/10.1021/acs.est.0c03211

Net, S., Sempéré, R., Delmont, A., Paluselli, A., Ouddane, B., 2015. Occurrence, fate, behavior and ecotoxicological state of phthalates in different environmental matrices. Environmental Science and Technology 49, 4019–4035. https://doi.org/10.1021/es505233b

US CPSC, 2018. Toxicity Review of DEHA.
